# Long-acting PEGylated recombinant human growth hormone (Jintrolong) for children with growth hormone deficiency: phase II and phase III multicenter, randomized studies

**DOI:** 10.1530/EJE-16-0905

**Published:** 2017-05-30

**Authors:** Xiaoping Luo, Ling Hou, Li Liang, Guanping Dong, Shuixian Shen, Zhuhui Zhao, Chun Xiu Gong, Yuchuan Li, Min-lian Du, Zhe Su, Hongwei Du, Chaoying Yan

**Affiliations:** 1 Department of PediatricsTongji Hospital, Tongji Medical College, Huazhong University of Science and Technology, Wuhan, Hubei, China; 2 Department of EndocrinologyThe Children’s Hospital, Zhejiang University School of Medicine, Hangzhou, Zhejiang, China; 3 Department of PediatricsThe First Affiliated Hospital of Zhejiang University, Hangzhou, Zhejiang, China; 4 Department of EndocrinologyChildren’s Hospital of Fudan University, Shanghai, China; 5 Department of EndocrinologyBeijing Children’s Hospital, Capital Medical University, Beijing, China; 6 Department of PediatricsThe First Affiliated Hospital of Sun Yat-Sen University, Guangzhou, Guangdong, China; 7 Department of EndocrinologyShenzhen Children’s Hospital, Shenzhen, China; 8 Department of PediatricsThe First Hospital of Jilin University, Changchun, Jilin, China

## Abstract

**Objective:**

We assessed the efficacy and safety of a weekly pegylated human growth hormone (PEG-rhGH) (Jintrolong) vs daily rhGH for children with growth hormone deficiency (GHD).

**Design:**

Phase II and III, multicenter, open-label, randomized controlled trials.

**Methods:**

108 and 343 children with treatment-naive GHD from 6 hospitals in China were enrolled in the phase II and III studies respectively. Patients in the phase II study were randomized 1:1:1 to weekly Jintrolong (0.1 mg/kg/week PEG-rhGH complex), weekly Jintrolong (0.2 mg/kg/week PEG-rhGH complex) or daily rhGH (0.25 mg/kg/week) for 25 weeks. Patients in the phase III study were randomized in a 2:1 ratio to weekly Jintrolong (0.2 mg/kg/week) or daily rhGH (0.25 mg/kg/week) for 25 weeks. The primary endpoint for both studies was height velocity (HV) increase at the end of treatment. Other growth-related parameters, safety and compliance were also monitored.

**Results:**

The phase II study established the preliminary efficacy, safety and recommended dose of Jintrolong PEG-rhGH. In the phase III study, we demonstrated significantly greater HV increases in patients receiving Jintrolong treatment (from 2.26 ± 0.87 cm/year to 13.41 ± 3.72 cm/year) vs daily rhGH (from 2.25 ± 0.82 cm/year to 12.55 ± 2.99 cm/year) at the end of treatment (*P* < 0.05). Additionally, significantly greater improvement in the height standard deviation scores was associated with Jintrolong throughout the treatment (*P* < 0.05). Adverse event rates and treatment compliance were comparable between the two groups.

**Conclusion:**

Jintrolong PEG-rhGH at a dose of 0.2 mg/kg/week for 25 weeks is effective and safe for GHD treatment and is non-inferior to daily rhGH.

## Introduction

Several long-acting recombinant human growth hormone (rhGH) preparations have been developed in recent years to improve patient compliance, adherence and long-term efficacy of rhGH treatment in patients with growth hormone (GH) deficiency (GHD). Nutropin Depot, the first long-acting rhGH formulation, was withdrawn from the market in 2004 because of manufacturing issues (1, 2, 3). Subsequently, a few novel long-acting rhGH preparations such as NNC126-0083, LB03002, PHA-794428 and VRS-317 have been developed and evaluated (3, 4, 5, 6, 7, 8, 9, 10, 11, 12). Among them, further developments for PHA-794428 was stopped due to high incidences of injection-site lipoatrophy after first injection (3, 10). Additionally, the efficacy and safety of long-acting rhGH preparations such as ACP-001, NNbib195-0092, GX-H9, HM10560A, MOD-4023 and VRS-317 in adults and children are currently being evaluated by ongoing phase II or III clinical studies (13).

Identification of an optimal long-acting rhGH regimen remains important for treating patients with GHD. PEGylated-rhGH (PEG-rhGH) (Jintrolong) is a long-acting rhGH prepared by conjugating a branched polyethylene glycol (PEG) molecule to amino groups of rhGH. PEGylation of rhGH can increase protein stability, reduce non-specific absorption and antigenicity, reduce renal clearance and extend the elimination half-life of rhGH (7, 8, 9). In a phase I pharmacokinetics study, 12 GHD children were treated with weekly 0.2 mg/kg of Jintrolong subcutaneous (s.c.) injection for 6 weeks after 4-week washout of previous 7-day 0.0286 mg/kg/day daily rhGH s.c. injections. Noticeably higher maximum plasma concentration (*C*_max_), significantly longer half-life (*t*_1/2_) and slower plasma clearance were associated with PEG-rhGH vs daily rhGH, indicating PEG-rhGH may be appropriate for long-term treatment (14). Further, weekly PEG-rhGH injections led to comparable serum IGF1 concentration change vs daily rhGH, and there was no burst release associated with weekly PEG-rhGH, suggesting a potentially long therapeutic action (14).

A phase II study was conducted to explore the applicability and appropriate dosage of Jintrolong in treating children with GHD. Based on this phase II study, a phase III study was subsequently conducted to further evaluate the short-term efficacy and safety of weekly 0.2 mg/kg/week Jintrolong vs daily 0.25 mg/kg/week rhGH in children with GHD.

## Subjects and methods

### Patients

For both the phase II and III studies, children with confirmed treatment-naive GHD from 6 hospitals in China were enrolled. Inclusion criteria were as follows: (1) GH peak concentration of <7.0 ng/mL in two different stimulation tests (only 2 different stimulation tests was performed for each patient); (2) bone age (BA) ≤9 years for girls or ≤10 years for boys, and ≥2 years delay compared to the patient’s chronological age (CA); (3) short stature (height standard deviation score (HTSDS) <−2 based on of the Chinese general population standard for age) (15) and height velocity (HV) <4.0 cm/year (HV standard deviation score (HVSDS) could not be used as one of the inclusion criteria due to the fact that there is no proper reference value for HV of Chinese children); (4) prepubertal status (testicular volume <4 mL in boys and breast development Tanner stage 1 in girls) and (5) >3 years of age. Patients diagnosed with multiple pituitary hormone deficiencies (MPHD) who met the inclusion criteria were enrolled only if their MP were well controlled with levothyroxine or glucocorticoids. Patients were excluded if they had chronic diseases (including hematological diseases or malignancies) or have participated in other clinical trials.

Both the phase II and III studies were designed, conducted and reported in compliance with the Declaration of Helsinki and were approved by the Ethics Committee of Tongji Hospital, Tongji Medical College, Huazhong University of Science and Technology, functioning according to the 3rd edition of the Guidelines on the Practice of Ethical Committees in Medical Research issued by the Royal College of Physicians of London. All patients or their guardian(s) provided written informed consent after the purpose and nature of all procedures used were fully explained. Registration numbers at ClinicalTrials.gov for the phase II and phase III studies are Nbib1342146 and Nbib1495468 respectively.

### Methods

Both phase II and III studies were multicenter, randomized, open-label, controlled clinical trials. Patients in the phase II study were randomized in a 1:1:1 ratio to weekly s.c. injections of PEG-rhGH (Jintrolong, GeneScience Pharmaceuticals, Changchun, China) (0.1 mg/kg/week), weekly PEG-rhGH (0.2/mg/kg/week) or daily rhGH (Jintropin AQ, GeneScience Pharmaceuticals) (0.25 mg/kg/week) for 25 weeks. The dosage of the PEG-rhGH referred to the weight of the PEG-rhGH complex. Patients in the phase III study were randomized in a 2:1 ration to weekly s.c. injections PEG-rhGH at a dose of 0.2 mg/kg/week or daily s.c. injections rhGH (0.25 mg/kg/week) for 25 weeks.

Block randomization was used for both of the studies, wherein a computer-generated randomization sequence in blocks was prepared using SAS9.2 PLAN by an expert biostatistician at the School of Public Health, Huazhong University of Science and Technology. 18 blocks (length 6) were used for the phase II study and 30 blocks (length 12) were used for the phase III study. For the phase II study, the 6 patients in each block were randomized 1:1:1 to receive weekly PEG-rhGH (0.1 mg/kg/week) (2 patients), weekly PEG-rhGH (0.2 mg/kg/week) (2 patients) or daily rhGH (0.25 mg/kg/week) (2 patients). For the phase III study, the 12 patients in each block were randomized 2:1 to receive weekly PEG-rhGH (0.2 mg/kg/week) (8 patients) or daily rhGH (0.25 mg/kg/week) (4 patients) so that more data on the efficacy and safety of PEG-rhGH could be gathered and also more patients could get the benefit of trying a new treatment. The randomization sequence was then sealed and given to a qualified printing company to manufacture scratch cards for random allocation, with one randomization number corresponding to one scratch card on which relevant information was printed. Treatment allocation corresponding to the randomization number was also printed and covered with foil on each card. Patients enrolled in the study acquired their own randomization numbers based on the order of their entry into the study. Doctors then scratched the foil on the card corresponding to the randomization number of each patient to reveal his/her treatment allocation and executed the corresponding treatment. During the study, neither the doctors nor the patients could select the randomization number or skip a number, and each patient has only one assigned number that was used and unchanged throughout the study.

All patients were assessed at baseline and at 4, 13 and 25 weeks after treatment initiation, in accordance with the guideline provided by Growth Hormone Research Society (GRS) perspective on the development of long-acting GH preparations (13). At each assessment, height and weight were monitored and blood samples were tested for serum IGF1, cortisol, free thyroxine (T_4_), thyroid-stimulating hormone (TSH), calcium, phosphate, lipids, glycated hemoglobin (HbAlc), insulin, fasting blood glucose, 2-h postprandial blood glucose, anti-hGH antibody levels, complete blood count, liver and renal function and homeostasis model assessment of insulin resistance (HOMA-IR). Of note was the fact that as the dates of each participating patient’s visitations could not be strictly controlled and enforced, the blood withdrawal at each assessment took place between 2 days before the last injection and 2 days later, since after each Jintrolong injection, the serum concentration of IGF1 increased steadily, reached a peak concentration 2 days after the injection and remained elevated for 7 days (14); therefore, the actual serum IGF1 concentration measured for each patient could be a peak or trough. Mid-parental height (MPH) ((father’s height + mother’s height)/2 + 6.5 for boys and (father’s height + mother’s height)/2 − 6.5 for girls), MPH standard deviation score (MPH SDS) ((patient’s MPH − average height of normal 18 years old of the same gender)/height SD of normal 18 years old of the same gender), corrected height SDS (HTSDS) (HTSDS-MPH SDS) were also evaluated. Urinalysis and electrocardiogram were also performed. In addition, safety was monitored to assess the possible side effects of GH treatment. No assay for neutralizing antibody was performed.

BA radiography was performed at baseline and week 25 and was read by a central laboratory at Tongji Hospital, using the standards of TW3 (16). Serum IGF1 and anti-GH antibody levels were measured at the laboratory in the Department of Pediatric Endocrinology and Metabolism Research Laboratory, Tongji Hospital. All other laboratory analyses were performed at treating hospitals. Serum IGF1 level was measured by enzyme-linked immunosorbent assay (ELISA) using Model 680 Microplate Reader (BioTek) with a coefficient of variation of 4.5%. The ELISA kit for IGF1 level measurement was manufactured by Diagnostic System Laboratories (Webster, TX, USA). Serum IGF1 concentration was transformed into SDS values using a normal healthy reference (provided by Diagnostic System Laboratories).

Serum anti-hGH antibody levels were measured by radioimmunoassay (Beijing North Institute of Biological Technology, Beijing, China) with a coefficient of variation of 1.8%. All other tests were performed at the primary hospitals.

Primary endpoint for the phase III study was annual HV increase at the end of the treatment. Annual HV was determined by change in height from baseline at each time point divided by the period of time between the 2 measurements. Secondary endpoints included HTSDS, IGF1 and BA. As there is no proper reference value for HV of Chinese children, HVSDS could not be used as one of the efficacy endpoints.

### Statistical analysis

All statistical analyses were performed using SAS 8.1 (SAS Institute, Inc., Cary, NC, USA).

Sample size for the phase II study was not statistically determined since it was an exploratory dosage-determination study, and the treatment efficacy of PEG-rhGH could not be ascertained before the study. In view of the difficulty in recruiting patients for the study due to limited number of children with short stature caused by endogenous GHD, and in accordance with the requirement of Good Clinical Practice in China (17), a sample size of 90 patients randomized in a 1:1:1 ratio to each of the 3 treatment groups was established. Allowing for a dropout rate of 20%, a total of 108 patients with 36 patients in each treatment group were planned for the phase II study.

Since the phase II study was an exploratory study for the purpose of establishing a proper dosage for PEG-rhGH and the efficacy of PEG-rhGH could not be determined prior to the study, the sample size for the phase II study was not calculated. A sample size of 108 was chosen according to the Provisions for Drug Registration published by China Food and Drug Administration (CFDA) in 2005 that dictates a sample size of at least 100 patients for a phase II study (18). For the phase III study, the non-inferiority margin for the primary endpoint, HV increase (cm/year) at the end of the treatment was set at −2 cm based on the results of the phase II study, assuming a common variance of 16. Assuming an α of 0.025 and a β of 0.15, a sample size of at least 165 patients (110 for the weekly PEG-rhGH group and 55 for the daily rhGH group) was needed to achieve a power of 85%. In order to further guarantee the robustness of the results and also to conform to the requirements of Chinese Provisions for Drug Registration (18), a sample size of 300 patients (200 for the weekly PEG-rhGH group and 100 for the daily rhGH control group) was established. Assuming a dropout rate of 20%, a total of 360 patients with 240 and 120 patients in the weekly PEG-rhGH and daily rhGH groups respectively, were planned for the phase III study.

The efficacy analysis used an intention-to-treat (ITT) approach on the population comprising all randomized patients who had at least one treatment and efficacy record (full analysis set (FAS). Missing data were imputed using the last-observation-carried-forward (LOCF) method. Safety analysis was performed on safety set (SS) including all randomized patients who had at least one treatment and safety record. For auxological measurement data, within-group differences before and after GH treatment were assessed using the paired *t*-test and Wilcoxon signed-rank test. Inter-group comparisons were performed using analysis of covariance (ANCOVA) with baseline as the covariate, taking into account the center effect. Enumeration data were compared using the chi-square test (the Cochran–Mantel–Haenszel (CMH) test) and the Fisher’s exact test. Data were expressed as mean ± s.d. with significance set at *P* < 0.05.

## Results

### The phase II study

108 patients were randomized in a 1:1:1 ratio to receive weekly s.c. injections of PEG-rhGH (0.1 mg/kg/week) (low-dose group (LD)), (0.2 mg/kg/week) (high-dose group (HD)) or daily rhGH (0.25 mg/kg/week). The FAS and SS populations included 97 and 98 patients respectively. The patients’ demographics and baseline characteristics were well balanced among the 3 groups (Table 1). Particularly, the percentages of patients with MPHD were comparable among the 3 groups, and the patients’ MPH and corrected HTSDS were also well balanced among the 3 groups (Table 1). Additionally, the percentages of patients entering puberty at baseline, weeks 4, 13 and 25 were comparable among the 3 groups (at baseline and week 4, 3.13% (1/32), 0% (0/31) and 5.88% (2/34) for LD, HD and daily rhGH respectively; at weeks 13 and 25, 3.13% (1/32), 3.23% (1/31) and 5.88% (2/34) for LD, HD and daily rhGH respectively).

**Table 1:** Patients baseline characteristics in the phase II study (FAS). Data are presented as mean ± s.d. or as *n* (%).

	**PEG-rhGH** 0.1 mg/kg/week (LD)	**PEG-rhGH** 0.2 mg/kg/week (HD)	Daily rhGH 0.25 mg/kg/week	P value
*n*	32	31	34	
Chronological age (year)	10.91 ± 3.31	11.75 ± 3.95	10.54 ± 4.05	0.4507^b^
BA (year)				
Male	6.19 ± 2.20	7.65 ± 2.42	6.16 ± 2.51	0.0518^b^
Female	5.51 ± 1.54	5.60 ± 1.50	4.81 ± 2.16	0.6046^b^
Sex, *n* (%)				0.4907^a^
Male	23 (71.88)	25 (80.65)	23 (67.65)	
Female	9 (28.3)	6 (19.35)	11 (32.35)	
Weight (kg)	20.50 ± 7.23	23.01 ± 7.01	20.18 ± 6.06	0.1947^b^
Height (cm)	110.18 ± 12.84	116.45 ± 11.69	110.42 ± 14.59	0.1050^b^
MPH (cm)	164.95 ± 8.53	167.32 ± 6.56	165.38 ± 6.74	0.3101^b^
HV (cm/year)	2.58 ± 0.87	2.86 ± 0.80	2.70 ± 0.92	0.4468^b^
IGF1 SDS	−2.44 ± 0.50	−2.13 ± 1.09	−2.33 ± 0.81	0.3480^b^
HTSDS	−4.84 ± 1.59	−4.50 ± 2.20	−4.48 ± 1.48	0.6519^b^
Corrected HTSDS	−5.02 ± 1.86	−4.62 ± 2.16	−4.55 ± 1.77	0.5701^b^
Pituitary gland MRI, *n* (%)				0.9721^a^
Normal	16 (50.00)	15 (48.39)	16 (47.06)	
Abnormal^c^	16 (50.00)	16 (51.61)	18 (52.94)	
IGHD/MPHD (*n/n*)	29/3	27/4	30/4	0.9210^b^

a Differences between the two groups were compared using bidirectional Cochran-Mantel-Haenszel (CMH)-*χ*^2^ test. ^b^Differences among the three groups were compared using the *t*-test or the *χ*^2^ test. ^c^Pituitary hypoplasia.

BA, bone age; FAS: Full analysis set; HTSDS, height standard deviation score; HV, height velocity; IGF1, insulin-like growth factor-1; IGFBP-3, IGF-binding protein-3; IGHD, isolated growth hormone deficiency; MPH, mid-parental height; MPHD, multiple pituitary hormone deficiencies; MRI, magnetic resonance imaging.

At week 25, the mean HV associated with LD, HD and daily rhGH was 11.63 ± 3.29, 12.65 ± 2.88, and 14.06 ± 3.98 cm/year respectively. Compared to baseline, all treatment arms were associated with significant HV increase (9.05 ± 3.57, 9.78 ± 3.08 and 11.36 ± 3.96 cm/year for LD, HD and daily rhGH respectively, *P* < 0.0001 for all) and HTSDS increase (Table 2). Both HD and daily rhGH led to significantly greater HTSDS increase compared to LD at week 13, week 25, while HD and daily rhGH treatment showed comparable efficacy (Table 2), indicating that the HD regimen may be more effective than the LD regimen.

**Table 2:** Efficacy comparison of the 3 treatment groups in the phase II study (FAS) (LOCF). All values are presented as mean ± s.d.

	**PGE-rhGH** 0.1 mg/kg/week (LD)	**PEG-rhGH** 0.2 mg/kg/week (HD)	Daily rhGH 0.25 mg/kg/week	Inter-group P valueb
*n*	32	31	34	
Height (cm)				
4 weeks	111.28 ± 12.81^a^	117.75 ± 11.58^a^	111.68 ± 14.51^a^	–
13 weeks	113.31 ± 12.69^a^	119.96 ± 11.25^a^	114.43 ± 14.13^a^	–
25 weeks	115.79 ± 12.59^a^	122.64 ± 10.96^a^	117.45 ± 13.70^a^	–
HTSDS				
4 weeks	−4.67 ± 1.59^a^	−4.29 ± 2.17^a^	−4.26 ± 1.49^a^	–
Change from baseline	0.18 ± 0.14	0.21 ± 0.10	0.22 ± 0.14	>0.05
13 weeks	−4.34 ± 1.56^a^	−3.93 ± 2.15^a^	−3.80 ± 1.53^a^	–
Change from baseline	0.50 ± 0.23	0.57 ± 0.22	0.68 ± 0.33	0.0070
25 weeks	−3.94 ± 1.52^a^	−3.49 ± 2.12^a^	−3.28 ± 1.60^a^	–
Change from baseline	0.90 ± 0.36	1.01 ± 0.39	1.20 ± 0.56	0.0063
IGF1 SDS^c^				
4 weeks	−1.99 ± 0.87^a^	−1.43 ± 1.26^a^	−1.69 ± 1.04^a^	–
Change from baseline	0.45 ± 0.64	0.70 ± 0.84	0.64 ± 0.60	>0.05
13 weeks	−1.66 ± 1.41^a^	−1.03 ± 1.46^a^	−1.39 ± 1.24^a^	–
Change from baseline	0.78 ± 1.32	1.10 ± 0.92	0.94 ± 0.86	>0.05
25 weeks	−1.65 ± 1.02^a^	−1.13 ± 1.23^a^	−1.24 ± 1.13^a^	–
Change from baseline	0.79 ± 0.89	1.00 ± 1.12	1.10 ± 0.96	>0.05

a *P* < 0.0001. Within-group comparisons before and after GH treatment were performed using the paired *t*-test or Wilcoxon signed-rank test. ^b^Inter-group comparisons were performed using analysis of covariance (ANCOVA) with F-statistics with the baseline data as the covariates, taking into account the center effect. ^c^Blood withdrawal at each assessment took place between 2 days before the last injection and 2 days after, since after each Jintrolong injection, the serum concentration of IGF1 increased steadily, reached a peak concentration 2 days after the injection and remained elevated for 7 days (14); therefore, the actual serum IGF1 concentration measured for each patient could be peak or trough.

FAS, full analysis set; HTSDS, height standard deviation score; IGF1, insulin-like growth factor 1; LOCF, last observation carried forward.

LD, HD and daily rhGH had comparable efficacy in improving the levels of IGF1 at week 25 (*P* > 0.05) (Table 2).

Over the 25 weeks of study, 53 of the 98 patients (54.1%) reported a total of 132 adverse events. There were 44 events reported by 17 patients in the LD group, 29 events by 16 patients in the HD group and 59 events by the daily rhGH group. 81 events (61.4%) in 42 patients were considered to be drug-related. Most events were considered mild in severity (126 of 132, 95.5%), only 5 (3.8%) were considered moderate (2 of each from LD and HD group, 1 from the daily rhGH group) and 1 event (0.8%) was considered severe (in the daily rhGH group).

### The phase III study

#### Patients

343 patients in the FAS were randomized to receive PEG-rhGH (0.2 mg/kg/week, *n* = 228) or daily rhGH (0.25 mg/kg/week, *n* = 115). The FAS, PPS and SS populations all included 343 patients.

The baseline characteristics were mostly balanced except that girls in the daily rhGH group had slightly more advanced BA, and that children in the PEG-rhGH group were shorter (Table 3). Additionally, the percentages of patients with MPHD were comparable between the 2 groups, and the patients’ MPH and corrected HTSDS were also well balanced between the 2 groups (Table 3). Finally, the percentages of patients entering puberty at baseline, weeks 4, 13 and 25 were comparable between the 2 groups (at baseline and week 4, 0% (0/228) and 0.87% (1/115)) for PEG-rhGH and daily rhGH respectively; at week 13, 0.88 (2/228) and 0.87 (1/115) for PEG-rhGH and daily rhGH respectively; and at week 25, 4.39% (10/228)) and 4.35% (5/115) for PEG-rhGH and daily rhGH respectively).

**Table 3:** Patients baseline characteristics and conditions in the phase III study (FAS). Data are presented as mean ± s.d. or as *n* (%).

	**PEG-rhGH** 0.2 mg/kg/week (*n* = 228)	**Daily rhGH** 0.25 mg/kg/week (*n* = 115)	***P* value**
*n*	228	115	
Chronological age (year)	11.30 ± 3.50	11.77 ± 3.60	0.2465^b^
Sex, *n* (%)			0.7958^a^
Male	187 (82.02)	93 (80.87)	
Female	41 (17.98)	22 (19.13)	
Weight (kg)	23.22 ± 6.41	24.73 ± 7.91	0.0777^b^
Height (cm)	116.33 ± 12.74	119.16 ± 12.55	0.0548^b^
MPH (cm)	166.71 ± 6.93	166.42 ± 6.40	0.7101^b^
BA (year)			
Male	7.27 ± 2.09	7.49 ± 2.04	0.4199^b^
Female	5.15 ± 2.23	6.38 ± 2.52	0.0501^b^
BA/CA	0.62 ± 0.13	0.63 ± 0.13	0.6491^b^
HV (cm/year)	2.3 ± 0.9	2.3 ± 0.8	0.9277^b^
IGF1 SDS	−1.86 ± 1.08	−1.74 ± 1.08	0.3381^b^
HTSDS	−4.52 ± 2.02	−4.46 ± 1.89	0.7676^b^
Corrected HTSDS	−4.18 ± 2.24	−4.06 ± 2.06	0.6302^b^
Pituitary gland MRI, *n* (%)			0.9662^a^
Normal	124 (56.88)	64 (56.64)	
Abnormal^c^	94 (43.12)	49 (43.36)	
GH peak concentration (μg/L)	2.55 ± 2.34	2.41 ± 2.38	0.596^b^
IGHD/MPHD	216/12	108/7	0.771^b^

a Differences between the two groups were assessed using bidirectional disorder CMH-*χ*^2^ or Fisher’s exact probability test. ^b^Differences between the two groups were assessed using the *t*-test. ^c^Pituitary hypoplasia.

BA, bone age; BA/CA, bone age/chronological age; HTSDS, height standard deviation score; HV, height velocity; IGF1, insulin-like growth factor-1; IGHD, isolated growth hormone deficiency; MPH, mid-parental height; MPHD, multiple pituitary hormone deficiencies; MRI, magnetic resonance imaging.

#### Height velocity (HV)

At week 25, the mean HVs for patients in PEG-rhGH and daily rhGH were 13.41 ± 3.72 cm/year and 12.55 ± 2.99 cm/year respectively. Both treatments were associated with significant HV improvement (11.15 ± 3.87 cm/year and 10.31 ± 3.14 cm/year increases at week 25 for PEG-rhGH and daily rhGH respectively, both *P* < 0.0001). Importantly, significantly greater HV was associated with PEG-rhGH treatment vs daily rhGH at week 25 (13.41 ± 3.72 cm/year vs 12.55 ± 2.99, *P* < 0.05). Further, significantly greater HV increase from baseline was also associated with PEG-rhGH vs daily rhGH at week 25 (11.15 ± 3.87 cm/year vs 10.31 ± 3.14 cm/year, *P* < 0.05).

#### Height standard deviation score (HTSDS)

**Figure 1:**
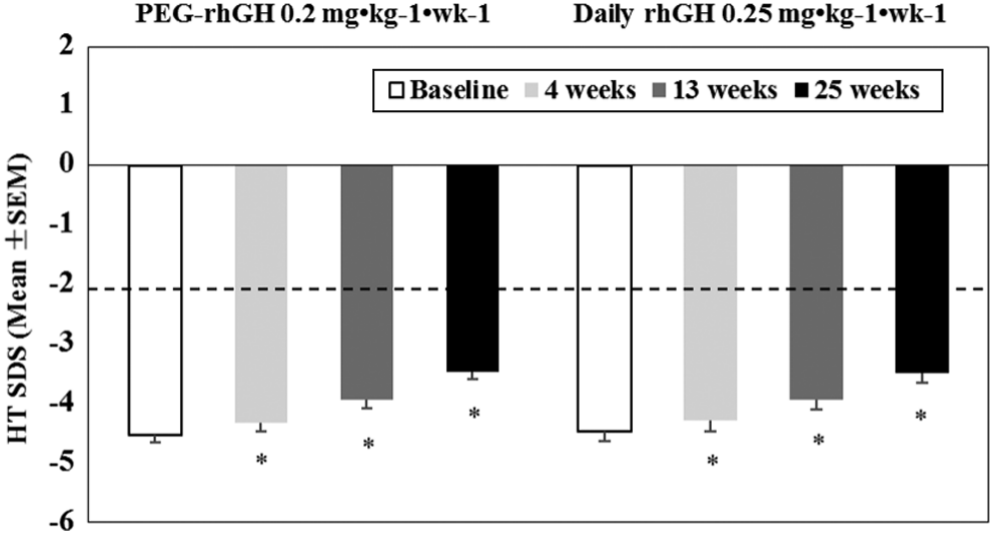
HTSDS for the PEG-rhGH and daily rhGH groups at baseline, weeks 4, 13 and 25 of the phase III study. The dotted line represents the lower reference limit.

At weeks 4, 13 and 25, HTSDS was significantly higher vs baseline with both PEG-rhGH and daily rhGH (*P* < 0.0001) (Fig. 1). In addition, significantly larger HTSDS increase was associated with PEG-rhGH treatment vs daily rhGH at week 4 (0.19 ± 0.11 vs 0.17 ± 0.10, *P* < 0.05), week 13 (0.58 ± 0.22 vs 0.53 ± 0.18, *P* < 0.05) and week 25 (1.06 ± 0.40 vs 0.98 ± 0.32, *P* < 0.05) (Fig. 1). There was a continuous convergence toward the normal range throughout the treatment period for both treatment groups (Fig. 1).

#### IGF1 SDS and BA/CA

**Figure 2:**
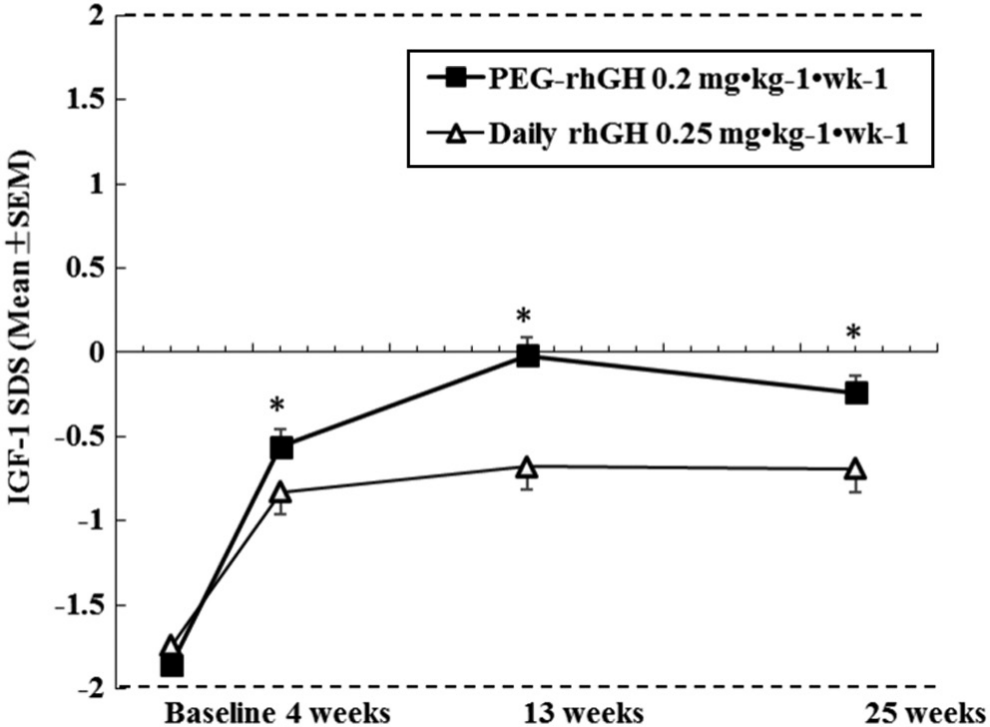
Mean IGF1 standard deviation score (SDS) for the PEG-rhGH and daily rhGH groups at baseline, weeks 4, 13 and 25 of the phase III study. The dotted lines represent the lower and upper reference limits.

Patients’ baseline serum IGF1 SDS were comparable between the two groups (−1.86 ± 1.08 and −1.74 ± 1.08 for the PEG-rhGH and the daily rhGH groups respectively). At week 25, significant increase in IGF1 SDS from baseline was associated with both groups. Throughout the treatment, serum IGF1 SDS was higher in the PEG-rhGH group vs daily rhGH group at all evaluation points (*P* < 0.01). Importantly, for both PEG-rhGH and daily rhGH treatments, IGF1 SDS reached a plateau at around week 13 and then gradually decreased (Fig. 2). 17 patients (7.5%) in the PEG-rhGH group and 5 patients (4.3%) in the daily rhGH group had elevated IGF-I levels above the upper limit of normal during the study (in the +2–4 SDS range) at 2 consecutive assessments, and there was no statistically significant difference between these two percentages (*P* = 0.267). Of note was the fact that the blood withdrawal at each assessment took place between 2 days before the last injection and 2 days after, since after each Jintrolong injection, the serum concentration of IGF1 increased steadily, reached a peak concentration 2 days after the injection and remained elevated for 7 days (14); therefore, the actual serum IGF1 concentration measured for each patient could be peak or trough.

The BA advancement after 25 weeks treatment matched CA advancement in PEG-rhGH group and daily rhGH group (0.53 ± 0.42 for PEG-rhGH and 0.59 ± 0.39 for daily rhGH). The mean BA/CA ratio increase from baseline at week 25 was comparable between PEG-rhGH and daily rhGH groups (0.03 ± 0.06 vs 0.03 ± 0.05, *P* > 0.05).

#### Analysis by peak GH

Based on GH peak concentration (≤5 μg/L or >5 μg/L) in the stimulation tests, patients were divided into two groups: moderate deficiency group (GH >5 μg/L) and severe deficiency group (GH ≤5 μg/L). In the PEG-rhGH group, the HV change from baseline was greater in the severe GHD group compared to the moderate GHD group (12.19 ± 3.48 cm/year vs 9.14 ± 3.12 cm/year, *P* < 0.001). Additionally, for patients receiving PEG-rhGH, compared to the moderate GHD group, the severe GHD group showed significantly greater height change (7.08 ± 1.87 cm vs 5.59 ± 1.33 cm, *P* < 0.001) and HTSDS change (1.11 ± 00.39 vs 0.9 ± 0.41, *P* = 0.005) but significantly less BA change from baseline (0.49 ± 0.40 year vs 0.67 ± 0.45 year, *P* = 0.003). Similar results were found in the daily rhGH group except that the server GHD group showed comparable BA change from baseline vs the moderate GHD group (data not shown).

#### Treatment compliance

Comparable treatment compliance was observed in patients receiving PEG-rhGH vs daily rhGH (96.9% vs 99.1%, *P* = NS).

#### Safety

Adverse events (AEs) were comparable between the PEG-rhGH and daily rhGH groups (37.32% vs 36.52%) and neither treatment resulted in any treatment discontinuation. PEG-rhGH treatment was associated with significantly higher peripheral edema (11.4% vs 1.74%, *P* = 0.002) and comparable rates of headache (1.75% vs 0.87%, *P* = 0.52) and injection-site reactions (0.88% vs 1.74%, *P* = 0.48) vs daily rhGH. Hypothyroidism was detected in 3 patients (1.4%) in the PEG-rhGH group and 1 patient (0.9%) in the daily rhGH group. One patient in the PEG-rhGH group had fasting hyperinsulinemia with normoglycemia. No severe adverse event occurred during the study.

In both treatment groups, fasting blood glucose levels, 2-h postprandial blood glucose, HbAlc, total cholesterol, triglycerides and HOMA-IR were in the normal range throughout the study (Table 4).

**Table 4:** Effects of GH treatment on safety parameters in GHD children in the phase III study ‘safety set’ (SS). All values are mean ± s.d., and the data is derived from the ‘safety set’.

	**Baseline**		**Week 13**		**Week 25**	
**Parameter**	PEG-rhGH (*n* = 228)	Daily rhGH (*n* = 115)	PEG-rhGH (*n* = 228)	Daily rhGH (*n* = 115)	PEG-rhGH (*n* = 228)	Daily rhGH (*n* = 115)
Insulin (U/L)	3.54 ± 2.96	4.44 ± 3.65*	5.99 ± 8.08	7.29 ± 8.21	6.46 ± 7.70	8.63 ± 10.21
Fasting blood glucose (mmol/L)	4.53 ± 0.60	4.64 ± 0.61	4.91 ± 0.69	4.96 ± 0.74	4.97 ± 0.66	5.03 ± 0.68
2-h postprandial blood glucose (mmol/L)	5.88 ± 1.36	5.87 ± 1.07	6.26 ± 0.96	6.10 ± 0.90	6.07 ± 1.00	6.03 ± 0.86
HbAlc (%)	5.38 ± 0.57	5.33 ± 0.60	5.46 ± 0.61	5.47 ± 0.74	5.46 ± 0.43	5.50 ± 0.44
HOMA-IR	0.11 ± 0.09	0.13 ± 0.11*	0.19 ± 0.23	0.23 ± 0.26	0.21 ± 0.24	0.28 ± 0.31*
Total cholesterol (mmol/L)	4.41 ± 0.96	4.47 ± 1.01	4.10 ± 0.95	4.07 ± 0.70	4.25 ± 0.97	4.23 ± 0.80
Triglycerides (mmol/L)	1.02 ± 0.76	0.92 ± 0.56	1.18 ± 0.71	1.09 ± 0.56	1.03 ± 0.74	1.03 ± 0.84

Differences between the two groups were compared using *t*-test.

* *P* < 0.05.

HbAlc, glycated hemoglobin; HOMA-IR, homeostasis model assessment of insulin resistance.

No subject in either group developed anti-GH antibodies during treatment when assessed at weeks 13 and 25; therefore, no assay for neutralizing antibody was performed.

## Discussion

Our phase II randomized study demonstrated that a weekly dosage of 0.2 mg/kg/week PEG-rhGH had better efficacy and comparable safety profile to a weekly dosage of 0.1 mg/kg/week. In phase III randomized study, we found that PEG-rhGH administered once weekly at a dose of 0.2 mg/kg/week was associated with greater increase in HV and serum IGF1 SDS level than daily rhGH at 0.25 mg/kg/week following 25 weeks of treatment in children with GHD. Our study only included treatment-naive patients with confirmed GHD and excluded other causes of short stature. Any possible residual effect of previous GH therapy could thus be excluded, and inclusion of only prepubertal subjects avoided the impact of puberty on HV.

The growth-promoting effect of our weekly PEG-rhGH was in line with results of other long-acting rhGH products (2, 11, 12). Importantly, our study showed greater efficacy in increasing HV, HTSDS and IGF1 SDS associated with PEG-rhGH vs daily rhGH at a lower weekly dosage than daily rhGH. Currently, we could not explain its underlying. Our PEG-rhGH’s molecular weight is 58–66 kDa (14). The treatment dosage of the long-acting weekly PEG-rhGH is not equivalent to a simple accumulation of daily dosage of the daily rhGH, and it has been suggested that for a long-acting rhGH formula, comparing its biological/physiological effects with those of daily rhGH would be more appropriate than a simple molar comparison (19). The long-term efficacy of PEG-rhGH in children with GHD needs to be explored further to assess its potential superiority.

There were some baseline differences between the two treatment groups that potentially could impact our outcome analyses; however, since ANCOVA incorporated these baseline characteristics as covariates, any bias resulting from the baseline differences would be minimized. One point worth noting is that the HV improvement did not occur at the expense of acceleration in skeletal maturation as reflected by the lack of acceleration in BA advancement relative to CA.

Achieving a satisfactory IGF1 profile is important for any long-acting rhGH preparation. The pegylated-rhGH manufactured by Novo Nordisk, NNC126-0083, was discontinued due to unsatisfactory weekly IGF1 profile: IGF1 response began subsiding approximately 3 days after administration of NNC126-0083 even at the highest tested dosage, and there was no evident dose-dependent IGF1 increase for AUC_0–168 h_ (3, 10). Serum total IGF1 level is currently the most commonly used biomarker for GH activity and prolonged supra-physiological IGF1 level should be avoided (3, 13). Although higher serum IGF1 in our study was associated with the PEG-rhGH group vs daily rhGH, the IGF1 SDS in the PEG-rhGH group was maintained within normal range throughout the 25-week treatment, and our PEG-rhGH did not initiate a worrisome increase of IGF1 SDS, rather, the higher serum IGF1 level associated with PEG-rhGH in our study suggests a greater per-mg efficacy (11). There is some concern about the possible association between elevated IGF1 and cancer (20); however, causality between elevated IGF1 and cancer has never been established (20). More importantly, our results showed comparable trends of change for IGF1 SDS after PEG-rhGH and daily rhGH injections. In fact, our phase III study revealed comparable percentage of patients in the PEG-rhGH group and the daily rhGH with elevated IGF-I levels above the upper limit of normal during the study (in the +2–4 SDS range) at 2 consecutive assessments (*P* = 0.267), this should alleviate the concern about escalating IGF-1 concentrations in patients receiving PEG-rhGH in view of our phase I study that revealed that post-Jintrolong injection serum concentration of IGF1 reached a peak concentration 2 days after the injection and remained elevated for 7 days (14). The GHD patients in both our phase II and III studies had lower-than-normal baseline IGF-1 concentrations (Fig. 2, Tables 1 and 3) and Jintrolong treatment could elevate their IGF-1 concentration into normal range, further indicating its efficacy. Overall, PEG-rhGH in our study did not initiate an IGF1 increase that should raise concern. IGF-1 level in great majority of the patients treated with PEG-rhGH were in the normal range, although a small percentage of subjects had elevated IGF-1 levels. In clinical practice, dose reduction would be recommended for these patients.

The spectrum and incidence of AEs were overall similar in patients who received PEG-rhGH and those receiving daily rhGH. No severe AEs were observed during the study. Our results showed that PEG-rhGH was safe and well tolerated. The safety profile of our PEG-rhGH was mostly consistent with previous reports of long-acting rhGH (9, 10, 11, 12). Our study did show higher incidence of peripheral edema associated with PEG-rhGH vs daily rhGH, while previous studies on LB03002 and Neutropin Depot did not report increased incidence of peripheral edema in pediatric patients with GHD (2, 11, 12, 21). In our study, most incidences of edema experienced by patients were mild and resolved themselves without treatment interruption. In addition, the higher incidence of edema associated with PEG-rhGH vs daily rhGH might correlate with the more effective growth-promoting effect of PEG-rhGH. Three patients in the PEG-rhGH group and 1 patient in the daily rhGH group had hypothyroidism during treatment, since it is well known that GH therapy could reveal previously undetected central hypothyroidism (defined by the serum free T_4_ falling into a subnormal range) and that thyroid function should therefore be monitored when GH therapy starts and whenever there is GH dosage adjustment (22), these patients possibly had undetected central hypothyroidism prior to enrolment.

No injection-site lipoatrophy was observed in our study. Injection-site lipoatrophy is an issue commonly encountered in some long-acting rhGH preparations (1, 22, 23, 24). The mechanism for lipoatrophy is not clear at this point; however, it was suggested to be unrelated to the high molecular weight of PEG but rather due to a direct lipolytic effect of GH on adipose tissue (24). Other evidence supporting that lipoatrophy is unrelated to PEG include the facts that injection-site lipoatrophy was reported in some non-PEG long-acting GH as well as daily GH (2) and that not all pegylated GH preparations lead to injection-site lipoatrophy (9). Injection-site lipoatrophy usually occurs after multiple injections at the same site (1, 23, 24). Our result of no injection-site lipoatrophy was consistent with the reports on LB030002 and NNC 126-0083 (9, 11). The lack of lipoatrophy seen in our patients receiving PEG-rhGH or daily rhGH injections may have been related to our efforts to avoid repeated injections at the same site and rapid subcutaneous absorption of PEG-rhGH.

No patients treated with PEG-rhGH developed anti-hGH antibody when assessed at baseline, weeks 4, 13 and 25. Some previous studies on long-acting rhGH formulations (none of which were pegylated) showed that some patients developed anti-hGH antibody (1, 11, 12). The relatively short duration of our study could contribute to this finding, as it has been reported that more patients on LB03002 treatment developed anti-rhGH antibody during the second year of the study vs the first year (12). Additionally, pegylated proteins/drugs usually have low immunogenicity/antigenicity (25, 26).

A recent position paper by the European Society of Paediatric Endocrinology (ESPE), GRS and the Pediatric Endocrine Society (PES) stated that despite the good safety record of GH therapy for approved indications at recommended dosage, continued monitoring of patients who received or are on rhGH is needed not only during the treatment but also in the years after the treatment (2). Also, the recently published GRS perspective on the development of long-acting GH preparations stated that such long-term monitoring is particularly important for long-acting rhGH preparations since long-acting rhGH preparations have very different pharmacokinetic and pharmacodynamic profiles when compared to daily rhGH (13).

A longer treatment period would strengthen our observations, especially in view of the ‘catch-up’ phenomenon in which higher catch-up growth during the first year of GH therapy is often accompanied by slower second-year growth rate (12). Therefore, our study did not address the question of whether the potent growth-promoting effect we observed with the PEG-rhGH could persist beyond 25 weeks, and whether its safety profile would change after 25 weeks. Studies longer than 1 year on Jintrolong PEG-rhGH’s efficacy and safety for GHD are currently underway, which also includes anti-rhGH antibody monitoring every 13 weeks. Another limitation of our study is that although we tested for the existence of anti-GH antibody, we did not test for anti-PEG antibody. It has been reported that anti-PEG antibody could accelerate the clearance of PEG-conjugated proteins and thus impact efficacy (27). Additionally, as mentioned before, as we could not strictly control and enforce the dates of each participating patient’s visitations, the actual serum IGF1 concentration measured for each patient could be peak or trough.

Finally, although our pre-clinical trial study in cynomolgus monkeys (Macaca fascicularis) did not find cellular vacuolation of the choroid plexus epithelial cells associated with repeated s.c. injection of PEG-rhGH, according to the recommendations of European Medicines Agency (EMA) regarding the use of pegylated drug products in the pediatric population (28), a study on 52-week recovery of cynomolgus monkeys (Macaca fascicularis) after 52 weekly s.c. injection of various doses of PEG-rhGH is currently ongoing in order to further understand the safety of long-term s.c. injection of PEG-rhGH.

In conclusion, PEG-rhGH at a dose of 0.2 mg/kg/week was effective and safe in treating GHD children up to 25 weeks and was non-inferior to daily rhGH treatment.

## Declaration of interest

The authors declare that there is no conflict of interest that could be perceived as prejudicing the impartiality of this study.

## Funding

Both the phase II and phase III studies were sponsored by GeneScience Pharmaceuticals Co., Ltd (Changchun, China).

## Author contribution statement

Xiaoping Luo was the principal investigator of the phase II and phase III studies who conceived the study. All authors participated in design of the study, patients’ enrolment and treatment, collecting, analysis and interpretation of the data. Xiaoping Luo wrote the first draft of the manuscript and all other authors make substantial contribution to its revision and gave their approval to the final version of the manuscript.
